# A Method to Realize Efficient Deep-Red Phosphorescent OLEDs with a Broad Spectral Profile and Low Operating Voltages

**DOI:** 10.3390/ma14195723

**Published:** 2021-09-30

**Authors:** Wei-Ling Chen, Shan-Yu Chen, Dun-Cheng Huang, Dian Luo, Hsueh-Wen Chen, Chih-Yuan Wang, Chih-Hao Chang

**Affiliations:** 1 Department of Electrical Engineering, Yuan Ze University, Taoyuan 32003, Taiwan; a608033@gmail.com (W.-L.C.); janet881115@gmail.com (S.-Y.C.); duncheng0609@gmail.com (D.-C.H.); 2 Institute of Lighting and Energy Photonics, National Yang Ming Chiao Tung University, Tainan 71150, Taiwan; dianluo.cop07g@nctu.edu.tw; 3 Raystar Optronic, Inc., Taichung 42881, Taiwan; hw.chen@raystar-optronics.com (H.-W.C.); cy.wang@raystar-optronics.com (C.-Y.W.)

**Keywords:** organic light-emitting diodes (OLEDs), phototherapy, exciplex, phosphorescent, broad spectral profile, power density

## Abstract

Organic light-emitting diodes (OLEDs) used as phototherapy light sources require sufficient spectral distribution in the effective wavelength ranges and low operating voltages. Herein, a double emitting layer structure consisting of a red-emitting Ir(piq)_2_acac and a deep-red Ir(fliq)_2_acac was designed to generate a broad electroluminescence spectrum. An efficient TCTA:CN-T2T exciplex system was used as the host of the emitting layer, facilitating effective energy transfer from the exciplex host to the red and deep-red phosphors. The materials used in the exciplex host were also used as the carrier transport layers to eliminate the energy barriers and thus increase the current density. The hole injection layer structures were varied to examine the hole injection capabilities and the carrier balance. The resulting optimized phosphorescent OLEDs with a broad spectral profile exhibit a 90% coverage ratio in the target ranges from 630 to 690 nm, together with a high peak efficiency of 19.1% (10.2 cd/A and 13.8 lm/W). The proposed device only needs 5.2 V to achieve a power density of 5 mW/cm^2^, implying that the device could be driven via two series-connected button cell batteries. These results illustrate the feasibility of our design concepts and demonstrate the realization of a portable and lightweight OLED phototherapy light source.

## 1. Introduction

Recently, organic light-emitting diodes (OLEDs) have gradually replaced TFT-LCD as the mainstream display technology. OLEDs are appealing for other applications, including in lightweight and flexible devices operating at high brightness under low power, suggesting the potential use of OLEDs in flexible, high-brightness and high-power applications such as phototherapy light sources and automotive taillights [1,2,3]. OLEDs also have some inherent advantages over other lighting technologies (e.g., inorganic LEDs), including low directivity, low operating temperature, transparency, non-fragility, flexibility/stretchability, and light weight [4]. Red OLEDs have attracted considerable attention because their emission wavelength ranges match the requirements of the aforementioned practical applications. In 2018, Jeon et al. presented a novel wearable photo-biomodulation (PBM) patch using a flexible red OLED as a phototherapy light source [5]. Their proposed red OLEDs with strong microcavity allow wavelength control in the 600–700 nm region while similar power density levels are maintained for each wavelength. The results demonstrated that this OLED-based PBM possesses superb in vitro wound healing effects due to the effective stimulation of fibroblast proliferation and the enhancement of fibroblast migration. Furthermore, their findings suggest that different wavelengths (e.g., 630–390 nm) may present different biologic responses depending on the energy level. In 2019, Lian et al. reported using flexible, top-emitting red OLEDs as light sources for photodynamic therapy to kill staphylococcus aureus [6]. By varying the thickness of the hole injection layer (HIL), the reported OLEDs can tune the emission peak from 669 to 737 nm to match the photosensitizer methylene blue. Results showed that more than 99% of bacteria were eliminated, indicating a huge potential for using red OLEDs to treat bacterial infections. In 2020, Choi’s group demonstrated a 6 μm-thick flexible OLED-based photonic skin which can be used for attachable phototherapeutics [7]. Since a thin Ag anode introduces the microcavity effect, the EL spectrum of the flexible OLEDs can be adjusted to a suitable wavelength range. Remarkably, up to 70% of artificial skin regeneration can be achieved by optimizing the irradiation interval.

Although the thin silver layer has the advantage of effectively modulating the spectrum due to the microcavity effect, OLEDs suffer pronounced energy loss if the emission peak is far from the emitter’s original emission peaks. In addition, most reported devices used a single emitter to match the target peak wavelengths [5,6]. However, the wavelength ranges for effective artificial skin regeneration are mainly from 630 to 690 nm [5]. The wavelength range of other related applications is also within or near this range. Since the fibroblast is not a single-wavelength artificial detector, we believe that a device with a broad spectral profile might produce better responses and have a wider range of applicability. Therefore, red OLEDs with a narrower spectral distribution caused by the microcavity effect should be avoided, and electroluminescence (EL) emission with a wider spectrum covering the effective ranges is preferred. On the other hand, the device’s light output should be strong enough to meet the requirement for energy treatments. In other words, the current density should be sustained at a high level to match actual usage requirements. Wearable PBM patches use a lightweight battery as the power source, and thus do not interfere with daily activity. Thus, the emitting layer (EML) of red OLEDs based on the exciplex-host systems should significantly boost the current density because of their controllable bipolar transport capability [8,9]. In addition, an ideal phototherapy light source should have a low operation voltage to allow for practical applications. This study does not perform biomedical research involving human subjects, but focuses rather on a device design that meets the criteria for phototherapy light sources. Herein, we propose a new deep-red OLED design with a broad spectral profile that simultaneously uses two efficient iridium phosphorescent emitters, enabling the device to reach high intensity in the target wavelength ranges, along with high device efficiency, high radiation power, and low operation voltages. The EL spectrum of the deep-red phosphorescent OLEDs (PhOLEDs) could achieve a broad spectral profile with a 90% coverage ratio in target ranges from 630 to 690 nm. The maximum efficiency of the devices reached 19.1% (10.2 cd/A and 13.8 lm/W). Moreover, the device only needs 5.2 V to achieve a power density of 5 mW/cm^2^.

## 2. Materials and Methods

### 2.1. Absorption and PL Spectrum Measurements

The absorption spectra were performed using a UV-VIS spectrophotometer (UV-1650PC, Shimadzu, Kyoto, Japan). The PL spectrum and lifetime measurements were characterized using a spectrofluorometer (FluoroMax-4, Horiba Jobin Yvon, Kyoto, Japan) with a Xenon arc lamp as the excitation source. The fluorescence spectrum of the tested samples was measured at room temperature, and the phosphorescence spectrum was measured at 77 K. The tested samples were placed in a Dewar with liquid nitrogen for phosphorescence. An excitation wavelength of 320 nm was adopted for PL measurement. Time-resolved photoluminescence (TRPL) was measured by monitoring the intensity decay at the peak wavelength using the time-correlated single-photon counting technique with a nanosecond pulsed LED (320 nm).

### 2.2. OLED Fabrication

Organic materials purchased from Shine Materials Technology were subjected to temperature-gradient sublimation in a high vacuum before use. After a routine cleaning procedure of ultrasonication of the indium tin oxide (ITO)-coated glass in deionized water and organic solvents, the ITO substrate was pretreated with plasma for 5 min. The organic and metal layers were deposited by thermal evaporation in a vacuum chamber with a base pressure of <10^−6^ Torr. Device fabrication was completed in a single cycle without breaking the vacuum. The deposition rates of organic materials and aluminum were, respectively, kept at around 0.1 nm/s and 0.5 nm/s. The active area was defined by the shadow mask (2 × 2 mm^2^). Current density-voltage-luminance characterization was carried out using two Keysight B2901A current source-measure units (Keysight, Santa Rosa, CA, USA) equipped with a calibrated Si-photodiode. The electroluminescent spectra of the devices were recorded using an Ocean Optics spectrometer (Ocean Optics 2000, Orlando, FL, USA).

## 3. Results

### 3.1. Exciplex Host and the Red Emitters

Generally, the mixture of some selected hole-transporting molecules (HTM) and electron-transporting molecules (ETM) could generate exciplex due to spatial proximity. The HTM and ETM respectively serve as the donor and acceptor in the exciplex system. Akin to the thermally activated delayed fluorescence (TADF), the exciplex can also form a small energy gap (Δ*E*_ST_) between the lowest singlet (S1) and triplet excited states (T1), allowing for reverse intersystem crossing (RISC) from triplet states to singlet states via environmental thermal energy [10]. The exciplex is considered a potential host for various emitters because of its great adjustable carrier transportability and effective host-guest energy transfer capability. The latter results from the guest’s ability to harvest both triplet and singlet excitons transferred from the exciplex host [8]. Theoretically, devices with an appropriate exciplex host in EML could reduce the operating voltage, adjust the device carrier balance, and improve device efficiency. When holes and electrons are injected into the EML, the deep red emitter may cause severe carrier trapping due to its narrow bandgap. Thus, red-emitting OLEDs generally possess high operation voltages [11]. Because exciplex is generated by mixing HTM and ETM, the exciplex host’s carrier transport capability is typically outstanding. Consequently, the EML of the device using the exciplex host will benefit the red-emitting devices operating at lower voltages.

**Figure 1:**
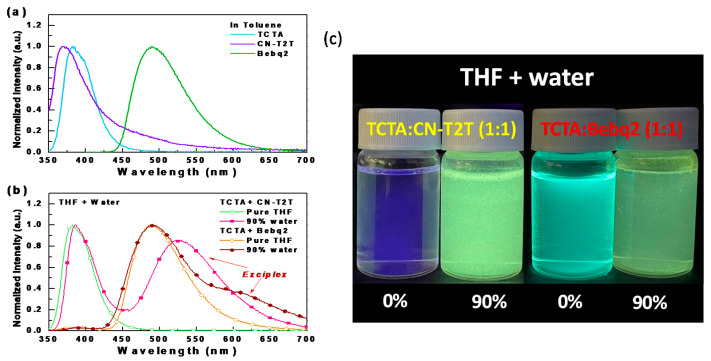
(**a**) Normalized PL spectra of the materials used in examining exciplex systems; (**b**) the tested samples in pure THF and with 90% water added; (**c**) the tested samples in THF with different water fractions under UV light illumination at room temperature.

To avoid the time-consuming traditional method of confirming exciplex formation, our previous study proposed a method to fast-screen exciplex formation by observing whether the mixture of the selected HTM and ETM shine under excitation in relatively high polar solvents [12]. 4,4′,4″-tris(carbazol-9-yl)triphenylamine (TCTA) was chosen as the donor to blend with two commonly used ETMs [13], bis(10-hydroxybenzo[h]quinolinato)beryllium (Bebq2) or 3′,3‴,3‴″-(1,3,5-triazine-2,4,6-triyl)tris(([1,1′-biphenyl]-3-carbonitrile)) (CN-T2T) [14,15], for the exciplex fast-screening. Figure 1a shows the PL spectra of the materials used in examining exciplex systems, while Figure 1b shows the PL spectra of the results of exciplex fast-screening. As indicated, the mixture of TCTA+CN-T2T sample in THF showed pure TCTA emission, while a strong exciplex emission of the sample in THF with 90% water fractions could be observed. On the other hand, the mixture of TCTA+Bebq2 sample in THF showed Bebq2 emission, while the sample in THF with 90% water fractions exhibited a relatively weak exciplex emission peak at about 608 nm. The spectral profile of the TCTA+CN-T2T exciplex is extensive and peaks at 527 nm, making it more suitable for the red or deep-red phosphors than the TCTA+Bebq2 counterpart. Figure 1c shows the two tested samples (D:A mixed ratio of 1:1) in pure THF and with 90% water added under UV light illumination at room temperature.

**Figure 2:**
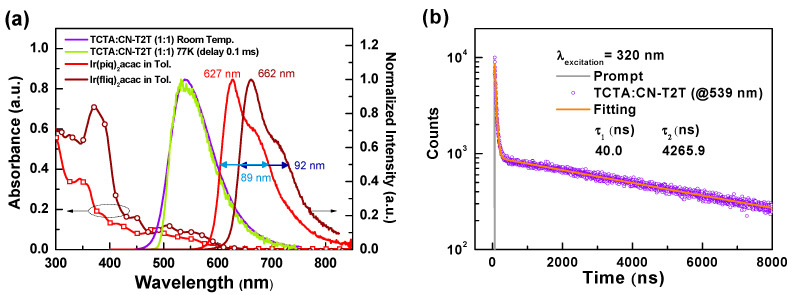
(**a**) Absorption and PL spectra of the TCTA:CN-T2T sample, Ir(piq)_2_acac, and Ir(fliq)_2_acac; (**b**) transient PL profiles of the TCTA:CN-T2T sample.

We fabricated the TCTA:CN-T2T (1:1) thin-film sample for PL measurements to determine the potential of the host-guest system. Figure 2a shows the PL and absorption spectra of bis(1-phenylisoquinoline)(acetylacetonate)iridium(III), (Ir(piq)_2_acac) and bis[1-(9,9-dimethyl-9*H*-fluoren-2-yl)-isoquinoline](acetylacetonate)iridium(III) (Ir(fliq)_2_acac) [16,17], together with the fluorescence and phosphorescence of the TCTA:CN-T2T thin-film sample. The TCTA:CN-T2T exciplex emission peak was recorded at 539 nm, which red-shifts to the longer wavelengths compared to the fluorescence of TCTA and CN-T2T. In addition, the close fluorescence and phosphorescence of the TCTA:CN-T2T indicates the Δ*E*_ST_ is extremely small, favoring the RISC from triplet to singlet via environmental heat [18]. Figure 2b shows the transient PL decay of the TCTA:CN-T2T sample. Again, the PL intensity decay curve of the excited state consists of prompt and delay decay segments, indicating that the delay term should result from the RISC. Based on the results of the PL spectra and the transient PL decay curves, TCTA mixed with CN-T2T could efficiently generate exciplex as expected. From Figure 2a, both Ir(piq)_2_acac and Ir(fliq)_2_acac have strong absorption ranging from 500 nm to 600 nm, overlapping with the PL spectrum of the TCTA:CN-T2T exciplex. The significant overlap with the absorption spectra of the selected red phosphorescent emitters implies favorable energy transfer from the exciplex host to the selected emitters [19]. On the other hand, the main peaks of Ir(piq)_2_acac and Ir(fliq)_2_acac are, respectively, at 627 nm and 662 nm. Both possess a wide spectral profile with clear vibronic features which cover most of the effective wavelength ranges for the biologic responses of human skin cells (i.e., 630 nm–690 nm) [5]. The full width at half maximum (FWHM) of Ir(piq)_2_acac and Ir(fliq)_2_acac were respectively estimated to be about 89 and 92 nm. These high FWHM values allow device designs to generate an ultra-wide EL spectral profile to fill the target wavelength ranges by designing a specific device architecture with these two efficient red and deep-red phosphors. We believe that uniform radiation intensity in this range can enhance absorption by different cells at different depths.

### 3.2. Device Architecture Design of Deep-Red PhOLEDs with Double EMLs

**Figure 3:**
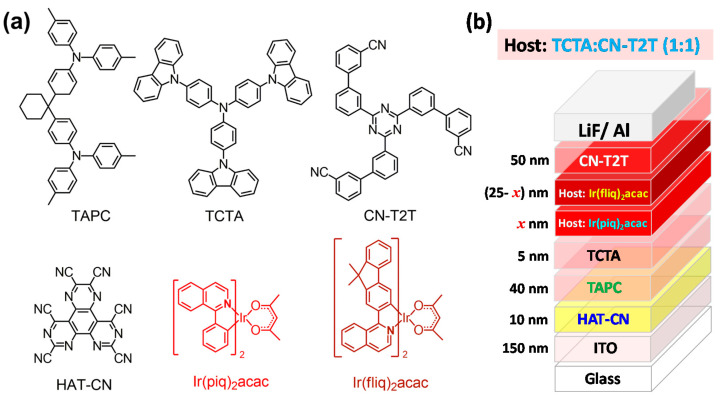
(**a**) Structural drawing of the materials and (**b**) schematic structures of the tested red OLEDs.

Herein, we describe the emitting layer (EML) of the proposed device architecture using an exciplex host to raise the current density and enlarge the carrier recombination zone. Based on the results mentioned above, the TCTA:CN-T2T was adopted as the exciplex host for the efficient red and deep-red emitters. In addition, the carrier transport layers adjacent to the EML used the same materials as the composed host to eliminate the energy barrier in order to raise the current density. Therefore, the hole transport layer (HTL) adopted TCTA while CN-T2T was used as the electron transport layer (ETL). In addition, our device architecture uses a composite structure of hole injection layers (HILs) consisting of dipyrazino[2,3-f:2′,3′-h]quinoxaline-2,3,6,7,10,11-hexacarbonitrile (HAT-CN) and 1,1-bis[(di-4-tolylamino)phenyl]cyclohexane (TAPC) [20,21], forming a step-wise hole injection into the TCTA layer. A double EML structure consisting of red and deep-red phosphors was designed to generate a wide-spectral profile to expand the coverage ratio of target wavelength ranges. The total thickness of the double EMLs was kept at 25 nm, and the thicknesses of both EML1 and EML2 varied correspondingly. The exciplex host used in both EMLs was a TCTA:CN-T2T mixture with a 1:1 ratio. The individual doping concentrations of both Ir(piq)_2_acac and Ir(fliq)_2_acac were carefully regulated to optimize device efficiency. Consequently, the designed devices were configured as ITO (150 nm)/HAT-CN (10 nm)/TAPC (40 nm)/TCTA (5 nm)/TCTA:CN-T2T:Ir(piq)_2_acac (46:46:8) (*x* nm)/TCTA:CN-T2T:Ir(fliq)_2_acac (48.5:48.5:3) (25–*x* nm)/CN-T2T (50 nm)/LiF (1.2 nm)/Al (120 nm), where the LiF and aluminum were respectively used as the electron injection layer and the cathode. The thicknesses of EML1 (Ir(piq)_2_acac)/EML2 (Ir(fliq)_2_acac) in the tested devices A–G are as follows: A: 25 nm/0 nm; B: 13 nm/12 nm; C: 11 nm/14 nm; D: 9 nm/16 nm; E: 7 nm/18 nm; F: 5 nm/20 nm; G: 0 nm/25 nm. Figure 3a shows a structural drawing of the materials used in the OLEDs, while Figure 3b shows the schematic structures of the tested deep-red PhOLEDs.

**Figure 4:**
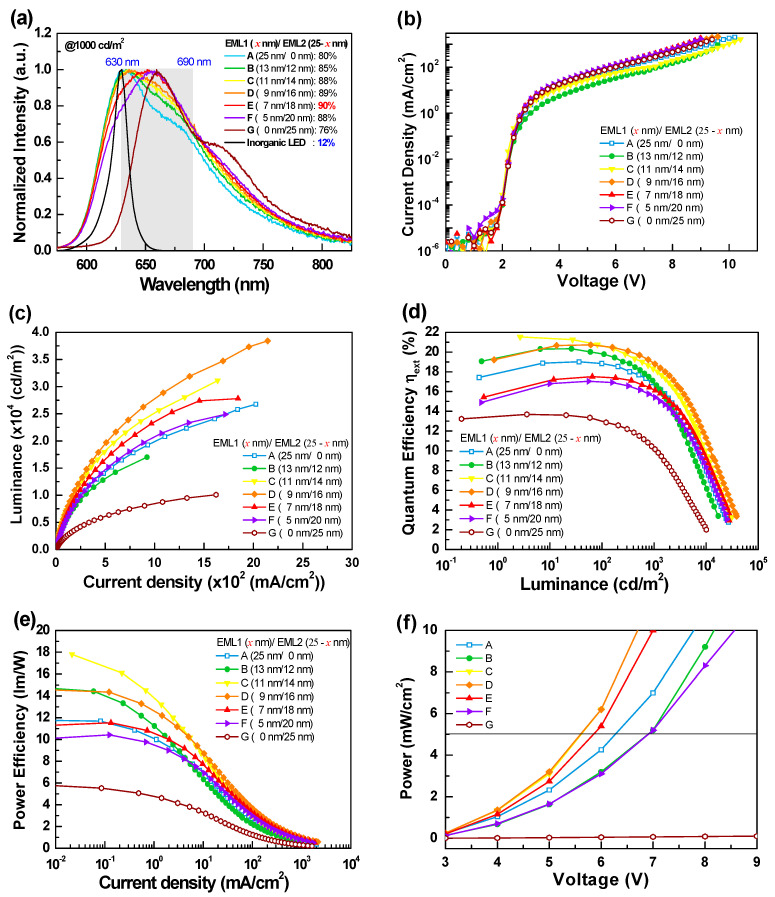
(**a**) Normalized EL spectra at a luminance of 10^3^ cd/m^2^, (**b**) current density- voltage (*J*-*V*) characteristics, (**c**) luminance-current density (*L*-*J*) characteristics, (**d**) external quantum efficiency versus luminance, (**e**) power efficiency versus current density, (**f**) power density versus voltage for devices A, B, C, D, E, F, and G.

Figure 4 shows the EL characteristics of the tested red OLEDs with different EML structures, while the corresponding numeric data extracted from the figures are shown in Table 1. As shown in Figure 4a, devices A and G with a single EML respectively presented pure Ir(piq)_2_acac and Ir(fliq)_2_acac emissions, indicating that the energy of the excitons formed in the EML were effectively transferred from the exciplex host to the emitter. In addition, the EL spectra of all devices presented broad spectral profiles composed of Ir(piq)_2_acac and Ir(fliq)_2_acac emissions, except for devices A and G, indicating that the carrier recombination zone could effectively form within the double EMLs [22]. Compared to device B, the Ir(fliq)_2_acac intensity increases along with the EML2 thickness. If we set the full target wavelength ranges (i.e., 630–690 nm) at 100%, the coverage ratio of the spectral profiles in the target wavelength ranges increased from 80% (device A) to 90% (devices E) and then decreased to 76% (device G). The EL spectrum of a 630 nm red inorganic LED was also added in Figure 4a for comparison, with a coverage ratio of only 12%. The respective FWHM measurements of devices A–G were 90, 94, 97, 100, 100, 100, and 88 nm. The strategy adopted here is usually used for white OLED designs to increase the EL spectrum covering all the visible wavelength ranges. Obviously, this design strategy is also valid for red OLEDs to create a wide spectral profile covering most of the target wavelength ranges.

Figure 4b shows the *J*-*V* curves of the tested devices. Devices A and G with a single EML exhibited similar current densities, indicating that Ir(fliq)_2_acac with a relatively lower energy bandgap would not cause more carrier trapping than Ir(piq)_2_acac. Comparing the devices with double EMLs, devices with a thicker EML1 (Ir(piq)_2_acac) exhibited relatively lower current densities. The current densities increased along with the EML2 thickness, possibly due to the positive influence of the lower doping concentration of Ir(fliq)_2_acac. As indicated, device D with both Ir(piq)_2_acac and Ir(fliq)_2_acac emissions exhibited the highest luminance of 38,420 cd/m^2^, while the lowest luminance of 10,085 cd/m^2^ was obtained in device G with a pure Ir(fliq)_2_acac emission. The external quantum efficiency and power efficiency of the tested devices are, respectively, shown in Figure 4d,e. From the efficiency curves, devices C and D exhibited the highest efficiencies, and the respective maximum efficiencies reached 21.5% (12.5 cd/A and 17.8 lm/W) and 20.7% (11.0 cd/A and 14.6 lm/W). Although device E only achieved a slightly lower peak efficiency of 17.5% (9.0 cd/A and 11.5 lm/W), it had the highest coverage ratio of 90% in the wavelength ranges (630–690 nm), making it the most qualified target device. Figure 4f shows the power curves of the devices. According to the literature, the effective radiation power density for application to human skin should exceed 5 mW/cm^2^ [6]. The operating voltage of device E recorded at 5 mW/cm^2^ was 5.9 V, obtained by using series-connected button cell batteries. Therefore, we could confirm that the exciplex host and the corresponding device architecture are conducive to operating under low voltage. This result demonstrates that this proposed strategy could produce a wide spectral profile with adequate operating voltages, thus meeting the requirements for a portable, lightweight PBM patch.

### 3.3. Structures of the HIL

Since device E exhibited adequate efficiency, the largest coverage ratio of the EL spectrum in the target wavelength ranges and a suitable operating voltage, device E was used for device optimization. To maintain the EL spectral profile, we only varied the HIL structures to investigate the device’s carrier balance condition. Consequently, the device architecture consisted of: ITO (150 nm)/HIL/TCTA (5 nm)/TCTA:CN-T2T:Ir(piq)_2_acac (46:46:8) (7 nm)/TCTA:CN-T2T:Ir(fliq)_2_acac (48.5:48.5:3) (18 nm)/CN-T2T (50 nm)/LiF (1.2 nm)/Al (120 nm). The tested HIL structures are summarized in Table 2. Device E was renamed device E1, and the corresponding EL characteristics were also included in this experiment for comparison.

**Figure 5:**
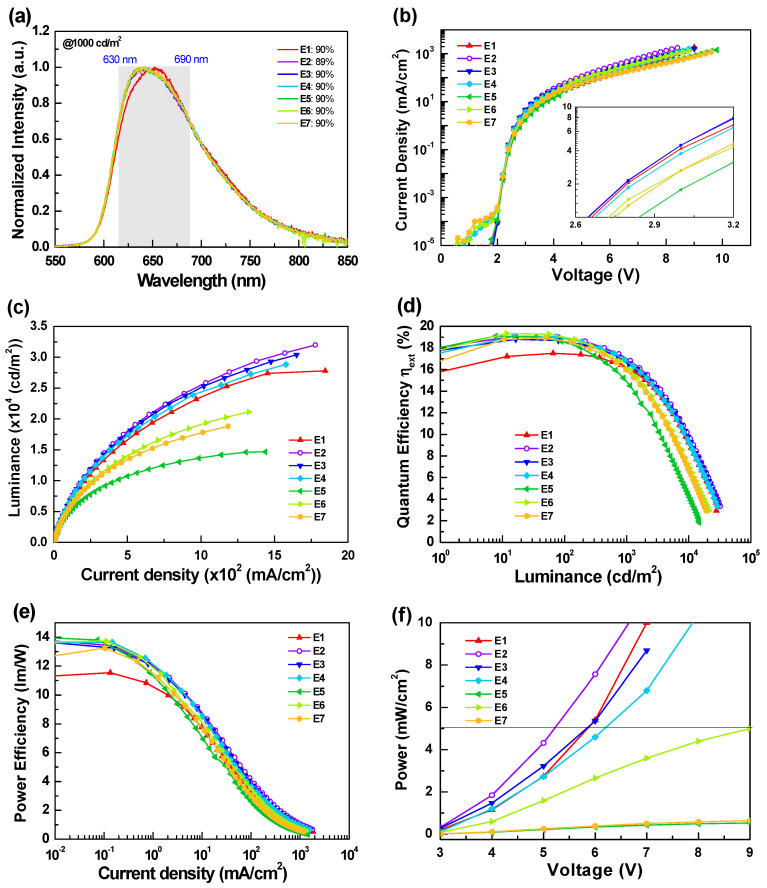
(**a**) Normalized EL spectra at a luminance of 10^3^ cd/m^2^, (**b**) current density-voltage (*J*-*V*) characteristics, (**c**) luminance-current density (*L*-*J*) characteristics, (**d**) external quantum efficiency versus luminance, (**e**) power efficiency versus current density, (**f**) power density versus voltage for devices E1, E2, E3, E4, E5, E6, and E7.

Figure 5 and Table 3 depict the EL characteristics of the tested red OLEDs with different EIL structures. From Figure 5a, the devices with newly designed HIL clearly showed different EL spectral profiles compared to device E1. Devices E2-E7 showed nearly identical EL spectral profiles, presenting a relatively stronger Ir(piq)_2_acac emission. Nevertheless, the FWHM of the EL spectra of devices E2-E7 remained large and ranged from 97 to 100 nm. In addition, their coverage ratios of the EL spectral profile in the target wavelength ranges (i.e., 630–690 nm) were as high as 90%, indicating that they are qualified from the EL spectrum aspect.

**Figure 6:**
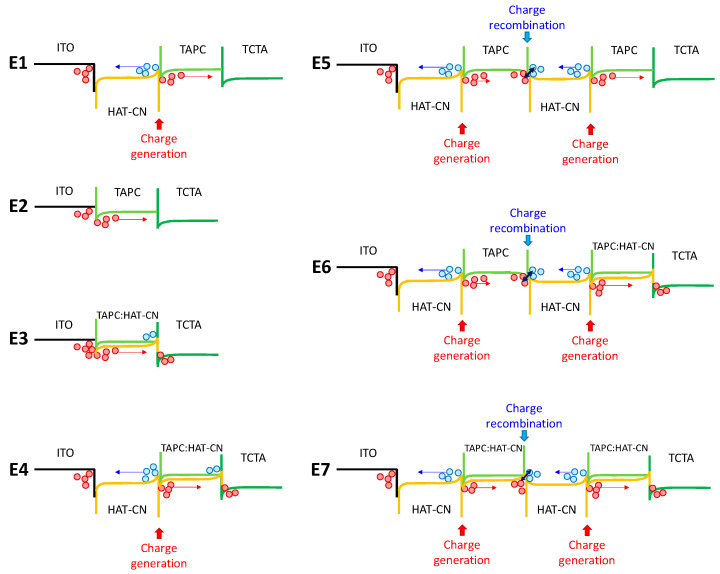
Schematic energy level diagram of the HIL structures of devices E1–E7 under bias.

Figure 5b shows the *J*-*V* curves of the devices, with current density comparison in the low voltage ranges (cf. Figure 5b inset) as follows: E2 ~ E3 > E1 >E4 > E6 > E7 > E5. Clearly, the multiple heterojunction pairs, such as HAT-CN/TAPC-based structures (i.e., devices E5–E7), hinder rather than increase current density. Furthermore, comparing devices E1–E4, devices E3 and E2 showed superior current densities than those with additional HAT-CN HIL. Unexpectedly, the HAT-CN/TAPC heterojunction did not improve the hole injection from the anode to the TAPC layer. Figure 6 presents a schematic energy level diagram of the HIL structures of the devices. As indicated, the weaker charge generation of the heterojunction of device E1 might result from the closed distance near the anode, thus interfering with the hole injection. A similar scenario was observed in device E4. In contrast, the work function of the ITO anode could be significantly increased through plasma treatment, matching the HOMO of TAPC and promoting smooth hole injection in devices E2 and E3. In general, a slight amount of HAT-CN doped in TAPC could benefit the current density. However, the slightly lower current densities obtained in device E4 indicate that the *p*-type doping (i.e., HAT-CN) could not countervail the negative influences resulting from the weaker charge generation. Only fewer holes could be injected into TAPC, as shown in device E4. On the other hand, the generated holes and electrons might cause recombination at the interface of the multiple heterojunctions pairs, as shown in devices E5–E7. The situation of mutual offset intensifies with the degree of heterojunction, leading to lower current densities. This phenomenon was opposite to the charge generation layer (CGL) structure observed in the tandem devices [23,24,25]. The HAT-CN/TAPC heterojunction is recognized as one of the most effective CGL structures. Ma et al. demonstrated that a device with HAT-CN/TAPC heterojunction pairs possessed a higher charge generation capability, indicating its effective charge generation capability [26,27]. We speculate that the applied electric field should influence the charge generation capability of the heterojunction structure once the structure is located adjacent to the electrode, which might reduce the charge generation capability. Figure 5c shows the device luminance. The slopes and the maximum luminance curves are similar to the current density discussed above.

Figure 5d,e show the external quantum efficiency and power efficiency curves. As indicated, devices E2–E7 showed similar peak efficiencies, all higher than that of device E1. Nevertheless, on closer inspection of the efficiency curves, devices E1–E4 sustained relatively higher efficiency when the luminance exceeded 1000 cd/m^2^, which is the practical range for PBM applications. The device maximum luminance values could also confirm their performance. The maximum luminance of devices E5–E7 were inferior to that of devices E1–E4, indicating that the device architectures of devices E5–E7 are not suitable for high luminance/power applications. In the overall evaluation of the tested devices, devices E2–E4 provide relatively satisfactory performance. Device E2, with its simplified device architecture, performed particularly well. The respective maximum efficiencies of devices E2 and E4 were respectively recorded at 19.1% (10.2 cd/A and 13.8 lm/W) and 19.1% (10.4 cd/A and 13.6 lm/W), while device E3 achieved a slightly lower peak efficiency of 18.8% (10.1 cd/A and 13.6 lm/W). The operation voltages of devices E2–E4 recorded at 5 mW/cm^2^ were respectively 5.2 V, 5.9 V, and 6.2 V. Clearly, device E2 only required 5.2 V operation voltage, thus meeting practical usage requirements, illustrating the application-appropriateness of this design. Compared with previously developed devices (target wavelength ranges 630–690 nm) in terms of the FWHM values of the EL spectrum, our designed OLEDs provide excellent device performance, including the most extended EL spectral profiles, low turn-on voltage, and adequate efficiency [28,29,30,31,32,33,34].

**Figure 7:**
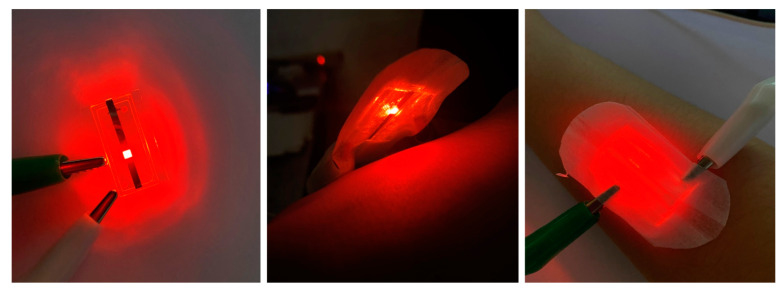
Schematic images of the PBM patch.

Figure 7 shows schematic images of the PBM patch. Button batteries can be used as the power source to increase patch portability. The manufactured device can be combined with breathable tape to allow users to move freely during treatment, greatly reducing inconvenience.

## 4. Conclusions

A wider EL emission spectrum covering the effective range of 630 nm to 690 nm is useful for improving OLED phototherapy effects. Furthermore, while providing sufficient power density (i.e., 5 mW/cm^2^), the operating voltage of the device needs to be as low as possible. A two-battery, lightweight wearable device would provide sufficient user convenience and freedom of movement. In this study, we propose a specific device design including double EML structures with red and deep-red phosphors, seeking to expand the device’s spectral profile to cover the target wavelength ranges. The coverage ratio of the EL spectrum reached 90% in the target wavelength ranges. In addition, an exciplex system, TCTA:CN-T2T, was introduced in the EML to facilitate effective host-guest energy transfer. The carrier transport layers adjacent to the EML were the same as the selected materials used to construct the exciplex, eliminating the energy barrier between the carrier transport layers and EML and thus enhancing the current density. As indicated, the maximum efficiency of the optimized device reached 19.1% (10.2 cd/A and 13.8 lm/W). The operation voltage of the device recorded at 5 mW/cm^2^ was 5.2 V, which could be obtained with two series-connected button cell batteries. These results illustrate the feasibility of our design concepts and provide a demonstration for the realization of OLED phototherapy.

## Figures and Tables

**Table 1:** EL characteristics of the tested red PhOLEDs with different thicknesses of the EML1 and EML2.

Device		A	B	C	D	E	F	G
EML1 (Ir(piq)_2_acac)/ EML2 (Ir(fliq)_2_acac)		25 nm/ 0 nm	13 nm/ 12 nm	11 nm/ 14 nm	9 nm/ 16 nm	7 nm/ 18 nm	5 nm/ 20 nm	0 nm/ 25 nm
External Quantum Efficiency (%)	*^a^*	19.0	20.3	21.5	20.7	17.5	17.1	13.7
	*^b^*	18.8	19.8	20.7	20.6	17.5	17.0	13.0
	*^c^*	16.7	16.9	18.3	18.9	16.2	15.5	10.3
Luminance Efficiency (cd/A)	*^a^*	9.0	11.0	12.5	11.0	9.0	8.1	4.2
	*^b^*	8.9	10.8	12.0	10.9	8.9	8.1	4.0
	*^c^*	7.9	9.2	10.6	10.0	8.3	7.3	3.2
Power Efficiency (lm/W)	*^a^*	11.8	14.8	17.8	14.6	11.5	10.4	5.8
	*^b^*	10.0	11.4	14.2	12.9	10.6	9.4	4.3
	*^c^*	6.4	6.2	8.7	8.8	7.3	6.5	2.2
V_on_ (V)	*^d^*	2.2	2.2	2.1	2.2	2.2	2.2	2.2
V (V)	*^e^*	6.3	6.9	5.6	5.6	5.9	6.9	-
L_max._ (cd/m^2^) [V]		26,763 [10.2]	17,014 [9.6]	31,116 [10.4]	38,420 [9.6]	27,799 [9.0]	24,883 [9.0]	10,085 [9.4]
FWHM (nm)	*^c^*	90	94	97	100	100	100	88
Coverage ratio (%)	*^c^*	80	85	88	89	90	88	76
CIE1931 coordinate (x, y)	*^b^*	(0.69, 0.31)	(0.69, 0.31)	(0.69, 0.31)	(0.69, 0.31)	(0.69, 0.31)	(0.69, 0.31)	(0.67, 0.32)
	*^c^*	(0.69, 0.31)	(0.69, 0.31)	(0.69, 0.31)	(0.69, 0.31)	(0.69, 0.31)	(0.69, 0.31)	(0.66, 0.33)

*^a^* Maximum efficiency. *^b^* Recorded at 10^2^ cd/m^2^. *^c^* Recorded at 10^3^ cd/m^2^. *^d^* Turn-on voltage measured at 1 cd/m^2^. *^e^* Operation voltage recorded at a power density of 5 mW/cm^2^.

**Table 2:** Structures of the deep-red PhOLEDs with different HIL structures.

Device	HIL Structure
E1	HAT-CN (10 nm)/TAPC (40 nm)
E2	TAPC (50 nm)
E3	TAPC: HAT-CN 10 wt.% (10 nm)/TAPC (40 nm)
E4	HAT-CN (10 nm)/TAPC: HAT-CN 10 wt.% (10 nm)/TAPC (30 nm)
E5	HAT-CN (6 nm)/TAPC (8 nm)/HAT-CN (6 nm)/TAPC (30 nm)
E6	HAT-CN (6 nm)/TAPC (8 nm)/HAT-CN (6 nm)/ TAPC: HAT-CN 10 wt.% (8nm)/HAT-CN (6 nm)/TAPC (22 nm)
E7	HAT-CN (6 nm)/TAPC: HAT-CN 10 wt.% (8nm)/HAT-CN (6 nm)/TAPC: HAT-CN 10 wt.% (8nm)/HAT-CN (6 nm)/TAPC (22 nm)

**Table 3:** EL characteristics of the tested deep-red PhOLEDs with different HIL structures.

Device		E1	E2	E3	E4	E5	E6	E7
External Quantum Efficiency (%)	*^a^*	17.5	19.1	18.8	19.1	19.1	19.3	18.9
	*^b^*	17.5	18.9	18.6	18.9	18.5	19.0	18.7
	*^c^*	16.2	17.0	16.7	16.8	14.9	16.3	15.9
Luminance Efficiency (cd/A)	*^a^*	9.0	10.2	10.1	10.4	10.6	10.5	10.2
	*^b^*	8.9	10.1	10.0	10.3	10.2	10.3	10.1
	*^c^*	8.3	9.1	9.0	9.2	8.2	8.8	8.6
Power Efficiency (lm/W)	*^a^*	11.5	13.8	13.6	13.6	14.0	13.7	13.2
	*^b^*	10.6	12.1	12.0	12.3	11.4	12.1	11.6
	*^c^*	7.3	8.6	8.4	8.4	6.6	7.9	7.4
V_on_ (V)	*^d^*	2.2	2.2	2.2	2.2	2.2	2.2	2.2
V (V)	*^e^*	5.9	5.2	5.9	6.2	9.0	-	-
L_max._ (cd/m^2^) [V]		27,799 [9.0]	31,996 [8.4]	30,385 [9.0]	28,823 [8.8]	14,681 [9.8]	21,148 [8.8]	18,817 [9.6]
FWHM (nm)	*^c^*	100	97	98	100	99	97	99
Coverage ratio (%)	*^c^*	90	89	90	90	90	90	90
CIE1931 coordinate (x, y)	*^b^*	(0.69, 0.31)	(0.69, 0.31)	(0.69, 0.31)	(0.69, 0.31)	(0.69, 0.31)	(0.68, 0.31)	(0.69, 0.31)
	*^c^*	(0.69, 0.31)	(0.68, 0.31)	(0.68, 0.31)	(0.68, 0.31)	(0.68, 0.31)	(0.69, 0.31)	(0.68, 0.31)

*^a^* Maximum efficiency. *^b^* Recorded at 10^2^ cd/m^2^. *^c^* Recorded at 10^3^ cd/m^2^. *^d^* Turn-on voltage measured at 1 cd/m^2^. *^e^* Operation voltage recorded at a power density of 5 mW/cm^2^.

## Data Availability

Not applicable.
